# Unraveling Exercise Addiction: The Role of Narcissism and Self-Esteem 

**DOI:** 10.1155/2014/987841

**Published:** 2014-10-28

**Authors:** Antonio Bruno, Diego Quattrone, Giuseppe Scimeca, Claudio Cicciarelli, Vincenzo Maria Romeo, Gianluca Pandolfo, Rocco Antonio Zoccali, Maria Rosaria Anna Muscatello

**Affiliations:** Section of Psychiatry, Department of Neurosciences, University of Messina, Via Consolare Valeria 1, 98125 Messina, Italy

## Abstract

The aim of this study was to assess the risk of exercise addiction (EA) in fitness clubs and to identify possible factors in the development of the disorder. The Exercise Addiction Inventory (EAI), the Narcissistic Personality Inventory (NPI), and the Coopersmith Self-Esteem Inventory (SEI) were administered to a sample of 150 consecutive gym attenders recruited in fitness centers. Based on EAI total score, high EA risk group (HEA *n* = 51) and a low EA risk group (LEA *n* = 69) were identified. HEA reported significantly higher total score (mean = 20.2 versus 14.6) on the NPI scale and lower total score (mean = 32.2 versus 36.4) on the SEI scale than LEA. A stepwise regression analysis indicated that only narcissism and self-esteem total scores (*F* = 5.66; *df* = 2; *P* = 0.006) were good predictors of days per week exercise. The present study confirms the direct and combined role of both labile self-esteem and high narcissism in the development of exercise addiction as predictive factors towards the risk of addiction. Multidisciplinary trained health care providers (physiatrists, psychologists, and psychiatrists) should carefully identify potential overexercise conditions in order to prevent the potential risk of exercise addiction.

## 1. Introduction

In the Substance-Related Disorders section, the Diagnostic and Statistical Manual of Mental Disorders, fifth edition, DSM-5 [1], includes only gambling disorder as form of addiction that does not involve ingestion of substance, reflecting evidence that this repetitive behavior activates reward systems as well as drugs of abuse [2, 3].

It is noteworthy that in clinical practice we observe a clustering of different excessive and repetitive behaviors, with symptoms that appear comparable to those produced by gambling, involving hedonistic (e.g., “sex addiction”) or nurturant motives (e.g., “exercise addiction,” “shopping addiction,” and “internet addiction”) [4]. Actually, these addictive behaviors, although showing strong neural similarities to substance addiction, are not included in any official recognized medical or psychological frameworks because there is not enough peer-reviewed evidence to establish diagnostic criteria [5].

Regular and moderate physical activity plays a lead role in the maintenance of health and in disease prevention. For instance, it can reduce the risk of cardiovascular diseases [6], diabetes [7], colon and breast cancer [8–10], and depression and anxiety [11, 12]. Moreover, adequate levels of physical activity will decrease the risk of a hip or vertebral fracture and help in weight control [13].

Exercise is a subcategory of physical activity that is planned, structured, and repetitive with the aim of improving or maintaining one or more components of physical fitness. Habitual exercise shows significant benefits for both physical and mental well-being in adults, children, and teenagers.

Even in mental disorders, future modern therapeutic approaches should include physical exercise as part of multimodal intervention programs aimed to improve psychopathology and cognitive symptoms [14]. Exercise may also be a novel treatment for drug addiction [15].

The term “exercise addiction” was first used to underlie the beneficial aspects of habitual exercise in contrast to drug or alcohol abuse or other self-destructive behaviors [16]. Exercise addiction was considered a “positive” addiction because of its beneficial effects on well-being, until it was clear that, in many cases, overtraining and overexercise were associated with increased susceptibility to injuries or with sociooccupational dysfunctioning. Morgan [17] labeled cases of extreme overuse of exercise as new forms of “negative” addiction. Exercise addiction could turn the positive psychosocial effects of regular physical activity into a detrimental activity when affected subjects experience overpowering drives. This conceptualization is in line with the theory of the long-term negative effect of any type of addiction [18], since addictions may alter the subjective experience of the self and are often seen as a failure of self-regulation.

Szabo reported that addicted exercisers could experience deprivation symptoms with strong adverse effects on subjective states and well-being [19].

It is almost well known that addictive behaviors do not develop abruptly; rather they evolve through a process made up of several stages. According to the theoretical model of behavioral addictions [20, 21], exercise addiction should include the following components: salience, when exercise becomes the most important thing, mood modification, occurring when people adopt a coping strategy to regulate emotions, tolerance, a physiological increase of the amount of exercise required to reduce craving, withdrawal, as manifested by anhedonia and anxiety when gym activity is suddenly reduced, conflicts between the addicted person and others, and relapse, the tendency to repeated reversions to earlier patterns of the activity.

Another important aspect to consider is the distinction between primary exercise addiction when the exercise itself is the main aim and secondary exercise addiction that is generally a consequence of an eating disorder and serves the purpose of weight control. Some authors argue that exercise addiction does not exist in absence of an eating disorder [22], whereas others suggest the hypothesis that exercise addiction is separate from eating disorders, although it may share some of concerns about body and performance [23]. Research about the association between exercise addiction and eating disorders has definitely shown conflicting results [24].

As other addictive behaviors, exercise addiction should also be differentiated from compulsions and impulse control disorders. Addicted subjects are egosyntonic and enjoy what they are doing, whereas obsessive-compulsive subjects are egodystonic and dislike their obsessions although they feel compelled by them [25, 26].

The prevalence of exercise addicted in general population is about 3% [27]. Among Italian adolescents a rate of prevalence of 8.5% of exercise addiction was found [28]. In French fitness room a prevalence rate of 42% was found [29].

The overall negative consequences of exercise addiction suggest the need to identify possible risk factors for the development of the disorder; among these factors, self-esteem in behavioral addictions was explored in cross-sectional and longitudinal studies that have shown an association between internet addiction and low self-esteem [30] but its direct role in development of exercise addiction has never been investigated [31].

Carter et al. suggested that trait anxiety and obsessive-compulsiveness were associated with a higher commitment to exercise and narcissism with greater physical activity. Narcissistic traits have been also found in substance-addicted adolescents [32], and high cooccurrence rates of substance and alcohol abuse and dependence have been found in adults with Narcissistic Personality Disorder [33]. Moreover, longitudinal studies have shown that Narcissistic Personality Disorder in adolescents could be a predictor of subsequent substance use disorder [34].

The aim of this study was to expand on these previous findings assessing narcissistic traits and self-esteem in a sample of gym attenders. We hypothesized that self-esteem and narcissism may have a role in the development of exercise addiction.

## 2. Method

### 2.1. Participants

150 consecutive gym attenders, recruited in fitness centers, were asked to participate in the study. Each participant was informed about the study design and provided a signed informed consent. The study was introduced to the participants as an investigation into attitudes and beliefs about exercise activity and personality. Subjects were asked to answer self-report questionnaires anonymously.

### 2.2. Measures

Data were collected using a sociodemographic questionnaire; we evaluated the frequency of physical activity assessing the number of the days in the week with more of 3 hours of exercise in the fitness center.

The Exercise Addiction Inventory (EAI) [35] was used to identify subjects at risk for exercise addiction. EAI is a self-report, six-item questionnaire with a five-point Likert response option ranging from 1 “strongly disagree” to 5 “strongly agree.” The measure is based upon the six components of exercise addiction according to Griffiths et al. [36]. EAI scores were used to categorize gym attenders in “high EA risk” and “low EA risk” groups.

The Narcissistic Personality Inventory (NPI) [37, 38] was used to estimate narcissistic components. NPI is a 40-item self-report questionnaire based on DSM-IV-TR criteria for Narcissistic Personality Disorder. It identifies seven factors (Authority, Exhibitionism, Superiority, Entitlement, Exploitativeness, Self-sufficiency, and Vanity) associated with narcissistic traits.

The Coopersmith Self-Esteem Inventory (SEI) [39] was used to assess self-esteem on the basis of attitudes toward oneself and others and personal interests. Respondents are asked to state whether 50 favorable or unfavorable aspects of a person are “like me” or “not like me.”

### 2.3. Statistical Analysis

Data obtained from the study underwent check and quality control and, subsequently, descriptive and inferential statistical analysis. Continuous data were expressed as mean ± S.D.* t*-test was used to compare age, numbers of days per week of exercise activity, self-esteem, and narcissistic components between groups; noncontinuous data were expressed as percentages and chi-square analysis was used to test gender distribution. Effect size was provided by using Cohen's* d* statistic and was considered small when being lower than 0.50, moderate when ranging from 0.50 to 0.79, and large when being equal to or greater than 0.80. All the variables that reached statistical significance underwent correlational analyses; a linear regression analysis was further performed in order to evaluate the association between narcissistic features, self-esteem, and exercises' characteristics. A stepwise method was used to select the explanatory variables based on analysis of variance. Statistical analyses were performed using Statistical Package for the Social Sciences—SPSS 21.0 software (SPSS Inc., Chicago, IL, USA).

## 3. Results

From a total sample of 150 gym attenders, 120 subjects completed the study. Based on EAI total score, a high EA risk group (HEA: *n* = 51, mean age = 29.7) was identified. Low EA risk group (LEA) was formed by 69 subjects (mean age = 32.2). The prevalence rate of EA risk in gym attenders was 42.5%; no gender differences in the rate of risk for exercise addiction were found (Table 1).

HEA subjects reported significantly lower SEI total score (mean = 32.2 versus 36.4) and higher NPI total score (mean = 20.2 versus 14.6) than LEA group, as shown in Table 2.

Effect sizes were large in NPI “Authority” and “Entitlement” scores, moderate in NPI “Narcissism,” “Self-sufficiency,” “Superiority,” “Exhibitionism,” “Exploitativeness,” and “Vanity” scores, and small in SEI total score.

All the variables that reached statistical significance (days per week exercise, as dependent variables, and self-esteem, narcissism total score, and three narcissism factors scores, as independent variables) were analysed in a linear regression model. As a block, the 5 predictors accounted for 15.4% of the total variance in exercise frequency (*F* = 2.537; *df* = 5; *P* = 0.039) (Table 3).

Furthermore, stepwise regression analysis provided two models: in the first one (Table 4) Self-esteem, Authority, Superiority, and Exploitativeness were excluded and only narcissism resulted in a predictor of exercise frequency (*F* = 6.2;  *df* = 1;  *P* = 0.015), accounting for the 7.9% of the variance; the second one (Table 5) indicated that narcissism and self-esteem as a block (*F* = 5.66;  *df* = 2;  *P* = 0.006) were good predictors of days per week exercise, whereas other scales did not give a significant additional contribution to the prediction of exercise frequency.

## 4. Discussion

The present study was designed to evaluate narcissistic traits and self-esteem in a sample of high EA risk subjects compared with EA low risk controls. The results obtained showed that specific narcissistic traits were associated with the risk for exercise addiction. High levels of narcissism could explain the drive for sport and exercise. This is congruent with previous findings showing that highly committed exercisers had substantially higher levels of narcissism with greater physical activity than less committed exercisers [40].

The role of narcissism in development of addiction has been addressed since Freud [41], who considered substance use as a narcissistic object choice in which substance itself represents an oral extension of the ego. In general, narcissism seems to be a core factor of addiction. The fulfilling of narcissistic drives is mediated by repetitive behaviors that assure omnipotence and provide protection against the potential lack of gratification or admiration.

The association between narcissism and addiction raises the following question: which is the object, the substance, or the activity to which the narcissistic subject should become addicted? According to Morf and Rhodewalt [42], in narcissistic persons, the focus of addiction might be on the grandiose view of self, not on the approval from others. If the admiration of other people is not the goal in itself, narcissism may be characterized by cognitive distortions that exaggerate the importance of the self, even without receiving confirmation from others. Within this context, narcissistic drives may lead to pursuing intrapsychic rather than interpersonal satisfactions. This attitude has been defined as “narcissistic myopia” [43], a condition in which social skills and interpersonal judgment are neglected, whereas only the desire for admiration is cognitively processed. In such context, narcissistic features (Superiority, Authority, and Exploitativeness) may become themselves addictive craving behaviors. Narcissistic subjects also seem highly susceptible to tolerance, since they continuously need to increase the search for claims and triumphs, and to withdrawal, since they show emotional symptoms when receiving something different from the admiration they seek.

Murray et al. [44] have explored the relationship between exercise identity and exercise dependence, according to the assumption that exercise identity promotes behaviors consistent with the perceived role of the exerciser. They found that “exercise beliefs,” a component of exercise identity, were significantly associated with the odds of experiencing dependence symptoms, whereby “exercise role identity,” the other component of exercise identity, was not significantly associated with the same odds. Moreover, the authors suggested the need for further research examining other factors possibly related to exercise addiction, such as identity, affect, self-esteem, and self-efficacy.

Our results showed that HEA subjects were characterized by lower self-esteem when compared with LEA subjects. This result extends to exercise addiction previous findings on the role of low self-esteem in the development of other addictions, as internet or online game addiction [45].

Low self-esteem was also found in an Italian sample of subjects affected by exercise dependence [46]. Hall et al. [47] argued that social prescribed perfectionism had a direct positive effect on exercise addiction and, as perfectionism is linked with contingent self-worth, labile self-esteem may mediate the relationship between unconditional self-acceptance and exercise dependence.

There are some limitations in the present study. First of all, the cross-sectional design does not allow considering changes over time. Another limitation is the relatively small sample size. Furthermore, to evaluate concomitant psychopathological symptoms, such as eating disorders or muscle dysmorphia, it would have been more accurate to perform a clinical interview. Finally, although the frequency of exercise may not be directly related to the risk for exercise addiction, since professional athletes exercise more often and longer than subjects at risk for exercise addiction, we selected “days per week” as the dependent variable in the regression analysis. Other models may have a stronger rationale to be tested.

## 5. Conclusions

The first aim of this study was the assessment of prevalence of the risk of exercise addiction among clients of fitness centers; congruently to previous data, we found high rates of prevalence: forty-two percent of the regular clients were high risk-exercise addicted according to EAI score. Nevertheless, despite the significant prevalence rates found in research, no specific diagnostic criteria for this condition were established. Since high and intense exercise levels, such as substances of abuse, may display their effect acting on reward pathways, it would be suitable to better understand this condition, taking into account possible vulnerability factors. According to previous studies that have indirectly and separately examined narcissism and self-esteem in behavioral addictions, the present study confirms the direct and combined role of both labile self-esteem and high narcissism in the development of exercise addiction as predictive factors towards addiction, once that regular exercise initiation occurs. Although it is well recognized that regular exercise is associated with a variety of positive outcomes, the early identification of a peculiar personality profile characterized by “addictive orientation” may have a role in discriminating those vulnerable subjects who are at risk for developing exercise addiction. Furthermore, it should be borne in mind that exercise, such as other rewarding behaviors, may present a potential misuse/abuse feature beyond physical, psychical, and social benefits. Multidisciplinary trained health care providers (physiatrists, psychologists, and psychiatrists) should carefully identify potential overexercise conditions in order to prevent the potential risk of exercise addiction.

## Figures and Tables

**Table 1:** Demographic characteristics and frequency of exercise activity.

	High EA risk group *n* = 51	Low EA risk group *n* = 69	*P* value
Age	29.7 (S.D. 7.1)	32.2 (S.D. 10.1)	.007
Gender			
Males	33	36	
Females	18	33	
Days per week exercise	4.71 (0.7)	2.79 (0.4)	<.0001
Number of years of exercise	10.19 (10)	10.76 (6.9)	

**Table 2:** Coopersmith Self-Esteem Inventory (SEI) and Narcissistic Personality Inventory (NPI) scores in high EA risk and low EA risk groups.

	High EA risk group (*N* = 51)	S.D.	Low EA risk group (*N* = 69)	S.D.	*P* value	Cohen's *d*
SEI total score	32.2	10.7	36.4	8.8	<.0001	0.4
NPI						
Narcissism	20.2	7.5	14.6	7.5	<.0001	0.7
Authority	4.7	1.8	3.1	2.3	.005	0.8
Self-sufficiency	3.3	1.5	2.5	1.4	.05	0.5
Superiority	2.4	1.3	1.5	1.1	.01	0.7
Exhibitionism	3.1	1.6	2.1	1.7	.051	0.6
Exploitativeness	3.4	1.6	2.3	1.7	.032	0.7
Vanity	2.8	1.4	1.9	1.6	.027	0.6
Entitlement	2.9	1.2	1.9	1.3	.007	0.8

**Table 3:** Linear regression analysis.

	Unstandardized coefficients		Standardized coefficients		
Dependent variable	*B*	S.E.	Beta	*t*	*P*
Days per ${\text{week}}^{\underline{\text{a}}}$					
(constant)	4.203	.606		6.939	<.0001
Self-esteem	−.029	.014	−.287	−2.177	.034
Narcissism	−.011	.045	−.079	−.244	.808
Authority	.096	.124	.178	.776	.441
Superiority	.090	.158	.102	.567	.573
Exploitativeness	.138	.137	.203	1.010	.317

${}^{\underline{\text{a}}}R=.439$; *R* ^2^ = .193; *F* = 2.537; *P* = .039.

**Table 4:** Stepwise regression analysis, Model 1. Excluded variables: Authority, Superiority, Exploitativeness, and Self-esteem.

	Unstandardized coefficients		Standardized coefficients		
Dependent variable	*B*	S.E.	Beta	*t*	*P*
Days per ${\text{week}}^{\underline{\text{a}}}$					
(constant)	3.141	.373		8.411	<.0001
Narcissism	−.043	.017	−.287	2.5	.015

${}^{\underline{\text{a}}}R=.314$; *R* ^2^ = .099; *F* = 6.250; *P* = .015.

**Table 5:** Stepwise regression analysis, Model 2. Excluded variables: Authority, Superiority, and Exploitativeness.

	Unstandardized coefficients		Standardized coefficients		
Dependent variable	*B*	S.E.	Beta	*t*	*P*
Days per ${\text{week}}^{\underline{\text{a}}}$					
(constant)	4.102	.574		7.151	<.0001
Narcissism	.041	.017	.297	2.434	.018
Self-esteem	−.027	.013	−.264	−2.159	.035

${}^{\underline{\text{a}}}R=.410$; *R* ^2^ = .168; *F* = 5.657; *P* = .006.
